# Sleeplessness

**Published:** 1860-02

**Authors:** 


### HALL'S JOURNAL OF HEALTH.

**Our Legitimate Scope is almost boundless: for whatever begets pleasurable and harmless feelings, promotes Health; and whatever induces disagreeable sensations, engenders Disease.**

WE AIM TO SHOW HOW DISEASE MAY BE AVOIDED, AND THAT IT IS BEST, WHEN SICKNESS COMES, TO TAKE NO MEDICINE WITHOUT CONSULTING A PHYSICIAN.

**Vol. VII.] FEBRUARY, 1860. [No. 2.**

### SLEEPLESSNESS.

A GENTLEMAN of superior culture once became deranged, and after a weary time, at a well-conducted asylum, was restored to mental health and remained perfectly well. One of the most striking observations he made in detailing the remembered portions of his history for the period including his aberration, was that the madness came on him by a slowly-increasing inability to sleep, and at its height, it seemed to him that he did not sleep at all; but from the very first day he could get a little sleep, the mind began to clear, and the two continued *pari passu* until complete recovery.

The experience of medical men of all countries is in striking accordance with the above. And if a fact so well established was generally known, many persons would be saved from the living death of hopeless lunacy, and a large number from the abhorrent crime of self-destruction.

Sleep is the great renovator of the brain. It is during its rest, of sleep, that it is nourished and invigorated; and without that food of rest in sleep, the mind can no more be sustained, than the body without food.

One of the very worst economies of time, is that filched from necessary sleep. The wholesale but blind commendation of early rising, is as mischievous in practice, as it is errant in theory. Early rising is a crime against the noblest part of our physical nature, unless it is preceded by an early retiring. Multitudes of business men in large cities count it a saving of time, if they can make a journey of a hundred or two miles at night by steamboat or railway. It is a ruinous mistake. It never fails to be followed by a want of general well feeling for several days after, if, indeed, the man does not return home actually sick, or so near it, as to be unfit for a full attention to his business for a week afterwards. When a man leaves home on business, it is always important that he should have his wits about him; that the mind should be fresh and vigorous, the spirit lively, buoyant, and cheerful. No man can say that it is thus with him, after a night on a railroad or on the shelf of a steamboat.

The first great recipe for sound, connected, and refreshing sleep, is physical exercise. Toil is the price of sleep.

To sleep well, a man must be regular in his hours of retiring and rising, and avoid sleeping in the day-time. Nature will take healthfully a certain amount of sleep, as she will take healthfully a certain amount of food, and no more; all over tends directly to disease and ends in premature death. In the desire to avoid eating too much, men have weighed their food, but some have eaten too little and died before their time in consequence of an error of judgment. There need be no mistake in this regard as to sleep in ordinary health, for if a man retires regularly after judicious and usual eating and exercise, he will, if let alone, be waked up by nature the very moment he has had enough of repose for the needs of the system. Instinct teaches the infant and the mere animal to cease taking aliment when they have had enough. Infants and animals never have dyspepsia, if let alone, for nature is the wise apportioner. Thus is it with sleep. Nature, herself sleepless, wakes us up the moment we have had enough, if we are not tampered with. Thus it is with men who live temperately and regularly; they wake up within five minutes of the same time morning after morning. If we go to sleep again, if we take a "second nap," nature is thwarted, and the result is, we go to sleep later the following night, sleep more unsoundly and later in the day; or if we get up early, we become insupportably sleepy during the day-time, and this goes on until we have no refreshing, sweet, connected sleep, day or night, when the general health begins to wane and the spirits droop. The laugh is less joyous; the countenance less cheerful; the eye less bright, and the road is downward! If at this phase of affairs medicines are given to promote sleep, it is only an artificial repose; it does not build up the system, but soon begins to clog the whole machinery, and in due time, the wheels of life stop forever. For let it be remembered, that every form of anodyne, whether of hop or poppy, such as morphine, laudanum, or paregoric, will, if continued beyond a very few doses, constipate the bowels, take away the appetite, torpify the liver, and derange the whole digestive machinery.

Labor, then, physical industry, temperance, and regularity, are the great panaceas for producing sound, healthful invigorating sleep. Various substitutes have been adopted to serve as temporary expedients, some of which may excite a smile, but all may be of greater or less avail.

1. Fix the thoughts, on getting into bed, on some one thing, vast and simple; such as a cloudless sky, or the boundless ocean, or the ceaseless goodness of the great Father of us all.

2. It has been said that sleep is promoted by lying with the head towards the north, and not by any means to the west, because of certain electric currents.

3. A writer recommends to commence rolling the eye-balls round the circuit of the eye, in the same direction, until sleep comes.

4. Another avers that a better plan is to place the head in a comfortable position, shut the mouth, and breathe through the nostrils only, making an effort to imagine that you see the breath going out all the time.

5. We have known, on the failure of all forms of anodynes, the gentle, continuous friction of the soles of the feet with a soft warm hand, to be admirably successful.

6. When persons are prevented from sleeping by a slight hacking cough, sleep is sometimes induced by having two pieces of muslin, say six inches by four, and three or four folds thick, to be used alternately thus: have a saucer at hand, half filled with alcohol, dip one of the cloths into it, then press it out, so as not to dribble, and lay it across the chest, the upper edge of the cloth ranging with the collar-bones, let it remain five minutes, then put on the other, alternating thus (by the nurse) with as little motion or noise as possible, the patient being on his back in the bed composed for sleep.

7. A French medical journal advises on retiring, to put five or six bits of sugar candy, as large as a hazelnut in the mouth, averring that before they are melted the desired effect will have been produced. This may avail in a case of simple sleeplessness, not as the result of any special disease. We would not advise such an expedient, for persons have been known to lose life by going to sleep with something in the mouth. If it is attempted at all, the candy should be placed between the cheeks and the gums, and the mouth kept resolutely closed.

The general rule is, that persons require seven hours of sleep in summer, and eight in winter. There are however occasional exceptions. Women require less sleep than men; possibly because they are less in the open air, the soporific effects of which are seen in infants speedily going to sleep when taken out of doors.

Children require more sleep than those in maturer life. Old people seem to require very little sleep, except in extreme age; but then it is rather a doze, or in short naps. Much of the credit given to elderly people for early rising is not deserved. They get up early because they can't sleep any longer; nature does not want any more, and they feel better when up and about than when in bed.

Napoleon the Great, seemed to require very little sleep, and he had a remarkable facility in going fast asleep at will. Pichegru said that during a whole year's campaign, he did not sleep more than one hour in twenty-four. We knew a man, named Paxton, who having been an engineer or pilot on a steamboat on the Mississippi, was not able on leaving his employment to sleep more than three hours out of any twenty-four for several years, but he died early.

We earnestly advise that all who think a great deal, who have infirm health, who are in trouble, or who have to work hard, to take all the sleep they can get, without medicinal means.

We caution parents, particularly, not to allow their children to be waked up of mornings; let nature wake them up, she will not do it prematurely; but have a care that they go to bed at an early hour; let it be earlier and earlier, until it is found that they wake up of themselves in full time to dress for breakfast. Being waked up early, and allowed to engage in difficult or any studies late and just before retiring, has given many a beautiful and promising child brain-fever, or determined ordinary ailments to the production of water on the brain.

Let parents make every possible effort to have their children go to sleep in a pleasant humor. Never scold or give lectures or in any way wound a child's feelings, as it goes to bed. Let all banish business and every worldly care at bed-time, and let sleep come to a mind at peace with God and all the world.

